# Right Coronary Artery Button Pseudoaneurysm After the Modified Bentall Procedure

**DOI:** 10.7759/cureus.40144

**Published:** 2023-06-08

**Authors:** Kaitlin A Nguyen, Suresh Keshavamurthy, Jeongae Yoon, Mohammed A Kashem, Yoshiya Toyoda

**Affiliations:** 1 Department of Surgery, Division of Cardiovascular Surgery, Temple University Hospital, Philadelphia, USA; 2 Department of Cardiothoracic Surgery, University of Kentucky, Lexington, USA; 3 Department of Anesthesiology, University of Maryland, Baltimore, USA

**Keywords:** pseudoaneurysm, bentall, pseudoknot, coronary ostial complication, modified bentall procedure, coronary button pseudoaneurysm

## Abstract

Anastomoses of the coronary buttons are the Achilles’ heel of the modified Bentall procedure (MBP) for the repair of the aortic root and ascending aorta. We present a rare case of post-MBP right coronary artery button pseudoaneurysm in a 30-year-old man. The contained leak, attributed to a pseudoknot in the polypropylene suture, was visualized via computed tomography angiography and transesophageal echocardiogram and repaired under deep hypothermic circulatory arrest.

## Introduction

When compared with the classical Bentall technique, the modified Bentall procedure (MBP) greatly reduces the risk of coronary anastomotic complications, with the reattachment of mobilized coronary arteries via the endo-button technique. We present a rare case of a pseudoaneurysm due to a suture pseudoknot at the site of the right coronary ostial anastomosis in a 30-year-old man.

This case was previously presented at the October 2022 International College of Angiology, Wading River, NY, and the September 2021 Metropolitan ACS, Philadelphia, PA.

## Case presentation

A 30-year-old morbidly obese (body mass index: 36.3 kg/m^2^) gentleman with a history of poorly controlled hypertension and a family history of aortic aneurysm and dissection presented to the Emergency Department with tearing chest pain radiating to the back, palpitations, diaphoresis, and dyspnea. The systolic blood pressure was elevated to 220 mmHg, and the patient reported having taken his last antihypertensive medications approximately one month prior. He denied the use of sympathomimetic substances.

Computed tomography angiography (CTA) showed a dilated ascending aorta and root measuring 5.3 cm × 3.5 cm with type A acute aortic dissection. The intimal tear involved the non-coronary and left coronary cusps proximal to the level of the commissure and extended distally to the level of the mid-ascending aorta. The aortic arch and the descending aorta were normal in size and without apparent dissection.

The patient was taken emergently to the operating room, where he underwent an MBP with a 29 mm St. Jude mechanical aortic valve conduit and re-implantation of coronary arteries. Intraoperatively, the surgeon noted an intense fibrotic reaction surrounding the ascending aorta, suggesting possible aortitis. Microscopic examination of the aortic tissue revealed changes consistent with dissection, and aortic valvular tissue displayed myxoid degeneration. Further genetic testing for connective tissue disease was deferred. The patient recovered uneventfully and was discharged home.

**Figure 1:**
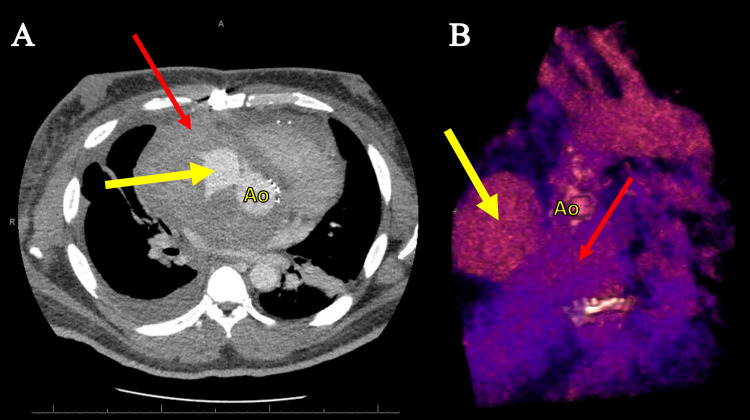
Preoperative computed tomography angiography of the chest. Large pericardial hematoma in retrosternal space (thin red arrow) with active extravasation (bold yellow arrow) from the right coronary artery re-implantation site: (A) axial view and (B) three-dimensional reconstruction.

On postoperative day 27, the patient presented again with dyspnea, palpitations, and chest pain. Transthoracic echocardiogram showed normal left ventricular function; moderate right ventricular dysfunction with mildly increased pulmonary artery systolic pressure; a well-seated, normally functioning mechanical aortic valve; and no pericardial effusion. Hemoglobin was 7.0 g/dL, which inadequately responded to transfusion. CTA revealed a large pericardial hematoma in the retrosternal space with active extravasation from the re-implantation site of the right coronary artery (RCA) and extrinsic compression of the RCA, superior vena cava, ascending aorta, and main pulmonary artery (Figure 1).

**Figure 2:**
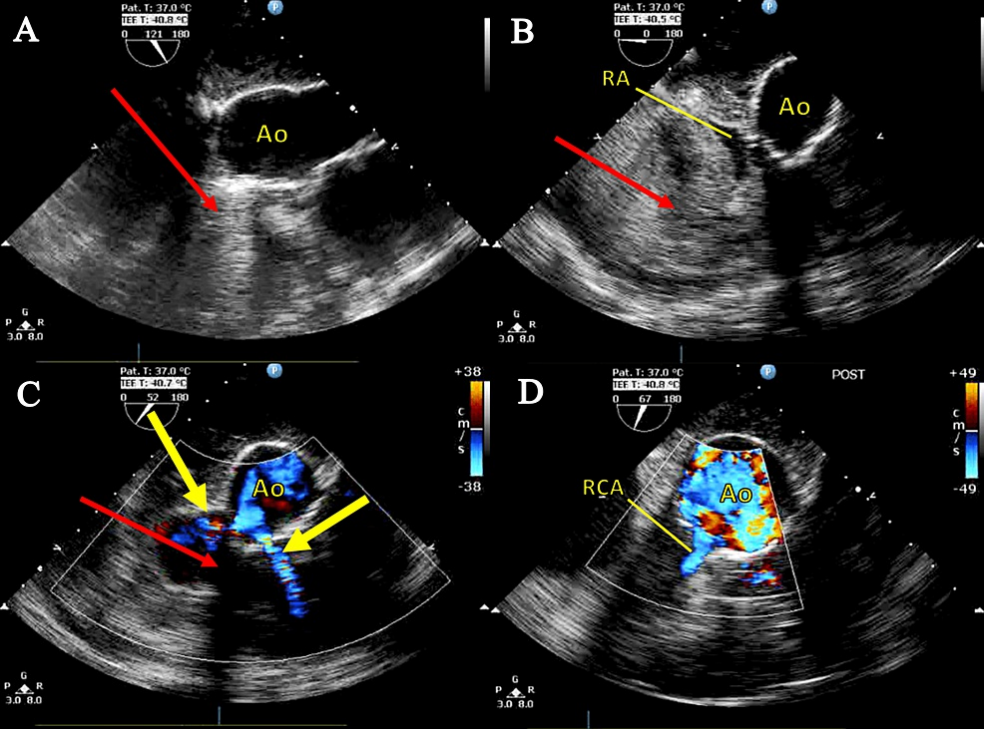
Intraoperative transesophageal echocardiogram images. Pseudoaneurysm with hematoma indicated by a thin red arrow. (A) Mid-esophageal aortic valve long-axis view: right coronary artery (RCA) button pseudoaneurysm anterior to the ascending aorta (Ao). (B) Upper esophageal ascending aortic short-axis view: pseudoaneurysm with compression of the right atrium (RA). (C) Color flow Doppler: two jets of blood flow (thick yellow arrows) visualized feeding pseudoaneurysm. (D) Color flow Doppler: post-repair of the RCA button and evacuation of the hematoma.

The patient was taken back to the operating room for urgent repair. Intraoperative transesophageal echocardiogram (TEE) displayed a large pseudoaneurysm with associated hematoma at the anterolateral aspect of the ascending aorta, with two high-velocity jets originating from the RCA button (Figure 2).

**Figure 3:**
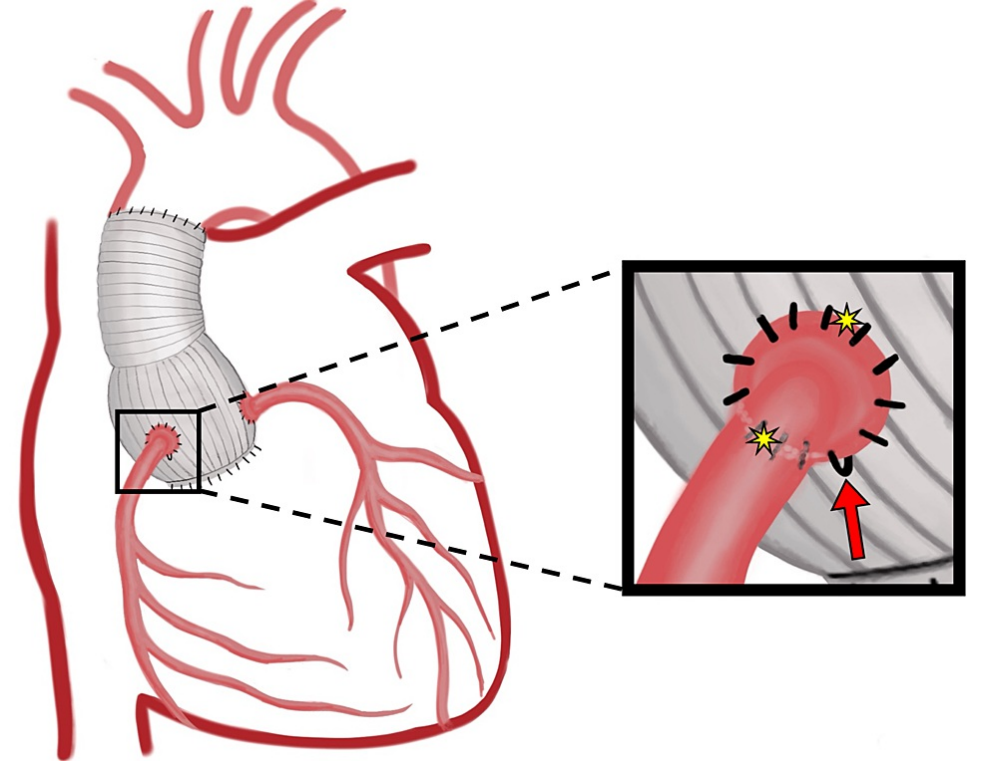
Illustration of the right coronary artery button before repair. Polypropylene pseudoknot at the six o’clock position (red arrow), and areas of active extravasation at the two and eight o’clock positions (yellow stars).

Due to the high risk of rupture of the pseudoaneurysm during re-entry, cardiopulmonary bypass was initiated via femoral cutdown and cannulation. After redo-sternotomy, dense adhesions were encountered from the innominate vein down to the root of the aorta, and the right atrium was enveloped in a thick hematoma. Deep hypothermic circulatory arrest at 18°C was initiated. After evacuating the hematoma around the aortic root, two leaks from the RCA button at the two and eight o’clock positions were repaired. An apparent pseudoknot on the polypropylene suture at the six o’clock position on the RCA button was straightened and tightened via nerve hooks (Figure 3). A separate suture was used to tighten the anastomosis and abolish the leaks. Antegrade cardioplegia was used to rule out further leakage. Recovery was uneventful and the patient was discharged home on postoperative day five.

## Discussion

Pseudoaneurysm following the MBP is a consequence of anastomotic failure at the proximal graft at the aortic annulus, the distal graft at the native ascending aorta, or the implantation sites of the coronary buttons. The advent of the MBP has significantly diminished the rate of these complications, with the incidence of pseudoaneurysm at the level of the coronary buttons reported at 1.5% and 3.3% [1-3]. Rare cases have been described, either in isolation or in combination with proximal or distal aortic graft dehiscence [4-8].

Pseudoaneurysms have been reported four months to 17 years after the index procedure [1,4-7,9-12]. In our patient, right coronary button dehiscence presented in the early postoperative phase.

Patients may be asymptomatic and discovered incidentally on routine follow-up or incidental imaging [4,7,9]. As in our case, patients present with dyspnea and chest pain [5,7,8,10-12]. Pulsating parasternal swelling has also been reported [5].

The pseudoaneurysm in our patient was attributed to a pseudoknot at the right coronary button anastomosis, discovered upon reoperation. The nature of the polypropylene suture material, a stiff monofilament with significant memory, contributes to pseudoknot formation. A technique to reduce the probability of pseudoknot formation involves pausing to disconnect the needle driver and straightening the suture when a quarter to a third of the anastomosis is complete.

In addition to iatrogenic injury, possible etiologies of pseudoaneurysms include poor tissue integrity due to connective tissue disease, erosion from infectious causes, graft-related porosity leading to seroma formation, uncontrolled hypertension, and the use of surgical sealants [8,9,11,13]. With a family history of aortic pathology and findings on gross and microscopic examination of the aortic tissue, connective tissue disease may have played a role in our patient’s anastomotic failure. The patient declined further diagnostic testing for relevant genetic diseases.

The strategy for repair of a post-MBP pseudoaneurysm is influenced by individual patient factors such as age, quality of native tissue, and size and location of the pseudoaneurysm. As in this case, open repair has been most frequently employed, but hybrid and transcatheter therapies may be considered as appropriate [4-11].

## Conclusions

The incidence of coronary anastomotic complications after aortic root replacement has decreased since the development and adoption of the MBP. Patients with connective tissue disease, infection, or inflammation may be at a higher risk of developing coronary anastomotic failure. Care must be taken to eliminate pseudoknot formation in polypropylene sutures and to ensure the fidelity of coronary anastomoses. The rare presentation of pseudoaneurysm and leaks can be acute, as in our patient, or delayed. Patients who present with post-MBP coronary complications and active extravasation require prompt surgical repair. Radiological imaging and intraoperative TEE are helpful adjuncts in diagnosis and treatment.
